# Comparison of lithium disilicate and feldspathic CAD-on systems in implant-supported crowns: a combined mechanical testing and FEA study

**DOI:** 10.1186/s40729-026-00701-6

**Published:** 2026-07-03

**Authors:** Elif Yeğin, Mustafa Hayati Atala

**Affiliations:** Department of Prosthodontics, Faculty of Dentistry, Istanbul Medeniyet University, Istanbul, Turkey

**Keywords:** CAD-on, Failure Load, Finite element analysis, Implant-supported restoration

## Abstract

**Background:**

The aim of this study was to compare the failure loads and stress distribution of two different CAD-on systems used for implant-supported crowns.

**Methods:**

Implant-supported crowns were designed with CAD/CAM software (Cerec InLab V15.0) and fabricated using the CAD-on technique (*n* = 12 per group): Group ZF (zirconia core veneered with feldspathic ceramic) and Group ZL (zirconia core veneered with lithium disilicate ceramic). Following cementation onto titanium abutments, the restorations were subjected to failure load testing using a universal testing machine. Simultaneously, 3D models of FEA were generated to evaluate Von Mises, Maximum Principal, and Minimum Principal stress distributions across the restoration, implant, abutment, and supporting bone. The mean failure loads were compared using the independent samples t-test (*p* < 0.05).

**Results:**

Group ZL exhibited significantly higher failure loads (4649.22 ± 733.42 N) compared to Group ZF (2085.91 ± 555.61 N) (*p* < 0.001). While stress concentrations were primarily located at the implant neck in both groups, the distribution on the abutments differed significantly. In Group ZF, delamination was the primary failure mode (100%), whereas Group ZL presented bulk fractures and abutment deformation.

**Conclusion:**

While the Group ZL exhibited significantly superior failure load, the veneer delamination observed in Group ZF could act as a protective failure mode. Although the FEA revealed that different veneer ceramics did not alter the stress distribution across the implant and the bone, the selection of restorative system should ideally balance high fracture resistance with the biomechanical safety of the implant components.

## Background

Dental implants have become the preferred therapeutic approach for the rehabilitation of tooth loss due to their high clinical survival rates [1, 2]. Despite this clinical success, dental implants are more susceptible to mechanical failures than natural teeth. Unlike tooth-supported restorations, the absence of a periodontal ligament leads to the direct transmission of occlusal loads to the supporting bone [3, 4]. This biomechanical limitation increases the concentration of stress within the restorative material, contributing to ceramic chipping, which is recognized as one of the most common technical complications in these restorations [5, 6].

In clinical practice, metal-ceramic restorations have a wide range of applications in oral rehabilitation cases [7]. However, to overcome the esthetic and biological limitations of these metallic frameworks, zirconia-based restorations have been increasingly preferred due to their high flexural strength, superior biocompatibility, and long-term clinical success [8, 9]. Zirconia frameworks are traditionally veneered through manual layering or heat-pressing to achieve better optical results [10]. The layering technique involves applying a mixture of ceramic powder and liquid onto the zirconia with a brush, followed by firing. Alternatively, the heat-pressing method is based on the lost-wax principle and produces a more homogeneous, less porous ceramic structure than manual layering [11]. Nevertheless, both methods are technique-sensitive and susceptible to clinical complications such as veneer chipping. This remains a major challenge in veneered zirconia restorations, occurring regardless of the implant location [10, 12]. While monolithic complete contour zirconia is used to prevent these failures [13], its high elastic modulus may lead to excessive stress transfer to the implant components and surrounding bone, potentially causing irreversible mechanical damage [12, 14].

Recently, CAD/CAM-based veneering techniques (CAD-on) have been developed as an alternative to traditional veneering and monolithic designs [15]. This method involves milling a zirconia framework and a glass ceramic veneer (such as lithium disilicate ceramic: LC or feldspathic ceramic: FC) separately [16]. These veneer materials have different microstructural characteristics: LC features a highly crystalline structure providing higher mechanical strength [17], whereas FC contains a higher glass phase, offering excellent optical properties but reduced mechanical resistance [18]. The two components are then bonded with a resin cement or fused using a specialized glass-ceramic, depending on the manufacturer’s protocol [16]. In the fusion process, high-frequency vibration is applied to a thixotropic glass-ceramic to achieve a precise and stable interface between the components before the firing [19]. The CAD-on technique employs high-quality, industrially manufactured ceramic blocks to minimize structural defects, optimizing the production process and reducing fabrication time [20]. Furthermore, a five-year clinical study reported high fracture resistance for three-unit posterior CAD-on fixed dental prostheses [19]. While investigating the mechanical behavior of such restorations is essential, finite element analysis (FEA) provides a reliable method to evaluate stress distribution within the implant system [21]. However, despite the clinical success of CAD-on systems, their fracture resistance and stress patterns in implant-supported restorations remain unclear. Specifically, the influence of different veneer materials and their bonding mechanisms on the biomechanical behavior of the implant-abutment complex has not been thoroughly investigated. Therefore, the aim of the current study was to compare the failure load and stress distribution of two different CAD-on systems for implant-supported crowns. The null hypothesis was that there would be no difference in the failure loads between the systems.

## Methods

### Preparation of test groups

The present study compared two different CAD-on systems for implant-supported crowns (*n* = 12 per group): Group ZF (zirconia core veneered with FC) and Group ZL (zirconia core veneered with LC). Technical specifications of the materials are listed in Table 1.

**Table 1:** Technical specifications and manufacturers of the materials

Material	Chemical composition (%)	Coefficient of Thermal Expansion (10^− 6^ K^− 1^)	Flexural strength (MPa)	Manufacturer
inCoris ZI; zirconia	ZrO_2_+HfO_2_+Y_2_O_3_ (≥ 99.0), Y_2_O_3_ (> 4.5 - ≤ 6.0), HfO_2_ (≤ 5), Al_2_O_3_ (≤ 0.5), Fe_2_O_3_ (≤ 0.3)	11	> 900	Sirona Dental Systems
IPS e.max CAD; Lithium disilicate glass ceramic	SiO_2_ (57–80), Li_2_O (11–19), K_2_O (0–13), P_2_O_5_ (0–11), ZrO_2_ (0–8), ZnO (0–8), Al_2_O_3_ (0–5), MgO (0–5), Colouring oxides (0–8)	10.2	360	Ivoclar Vivadent
Vita Mark II; feldspathic ceramic	SiO_2_ (56–64), Al_2_O_3_ (20–23), Na_2_O (6–9), K_2_O (6–8), CaO (0.3–0.6), TiO_2_ (0-0.1)	9.4	136	Vita Zahnfabrik
IPS e.max CAD Crystall./Connect; fusion glass ceramic	Water, butandiol and zinc chloride (11–30), Powder (70–90); SiO_2_ (50–65), Al_2_O_3_ (8–22), Na_2_O (6–11), K_2_O (4–8), ZnO (1–3), other oxides (5-17.5), pigments (0.1-3)	9.5	160	Ivoclar Vivadent

Twenty-four standard bone-level implants (Ø 4.1 mm; L 12 mm; Hager & Meisinger GmbH, Germany) were vertically positioned within plastic rings and secured using autopolymerizing acrylic resin (Vertex-Dental BV, Zeist, Holland). Subsequently, cement-retained abutments with a 5 mm diameter and 6 mm height (Hager & Meisinger GmbH, Germany) were torqued to the implants at 35 Ncm.

### Fabrication of the restorations

Following optical impressions (InEos Blue; Sirona Dental Systems GmbH, Bensheim, Germany), crown designs were performed (Cerec InLab V15.0) according to the morphology of a mandibular first molar. A multilayer design and “splint mode” were selected, maintaining a core thickness of 0.6 mm. The occlusal thickness was standardized at 1.5 mm at the central fossa and 2.0 mm at the cusps. Using a computer-aided milling system (InLab MC XL; Sirona Dental Systems GmbH, Bensheim, Germany), 24 zirconia cores, 12 LC (IPS e.max CAD; Ivoclar Vivadent, Schaan, Liechtenstein) and 12 FC (Vita Mark II; Vita Zahnfabrik, Bad Sackingen, Germany) veneers were produced. The zirconia cores were sintered at 1500 °C for 90 min (InFire HTC speed; Sirona Dental Systems, GmbH, Bensheim, Germany).

In the ZL group, a fusion glass-ceramic (IPS e.max CAD Crystall./Connect; Ivoclar Vivadent, Liechtenstein) was employed to connect the zirconia cores with the LC veneers. Following the removal of excess fusion material, the restorative complex was subjected to a combined crystallization-glazing process (Vita Vacumat 6000 M; Vita Zahnfabrik, Germany). In the Group ZF, the zirconia surfaces were airborne-particle abraded using 50 μm Al_2_O_3_. The FC veneers, once glazed (Vita Akzent Plus; Vita Zahnfabrik, Germany), were conditioned with 4% hydrofluoric acid (Bisco Porcelain Etch, Bisco Dental Products, USA) for 60 s, followed by silanization (Monobond Plus; Ivoclar Vivadent, Liechtenstein). Final connection was achieved using a dual-cure resin-based cement (Panavia F 2.0, Kuraray Medical Inc., Japan), followed by light-polymerization of all margins (Table 2). 

**Table 2:** Components and surface treatment protocols of the groups

Groups	Core	Veneering ceramic	Surface Treatment	Fusion material
ZF	Zirconia (inCoris ZI, Sirona Dental Systems, GmbH, Bensheim, Germany)	Feldspathic ceramic (Vita Mark II, Vita Zahnfabrik, Bad Sackingen, Germany)	-Sandblasting (50 μm Al_2_0_3_) to the zirconia cores -Etching veneers with 4% hydroflouric acid (Bisco Porcelain Etch, Bisco Dental Products, Schaumburg, IL, USA) - silanization of veneers (Monobond Plus, Ivoclar Vivadent, Schaan, Liechtenstein)	Dual-cured resin based cement (Panavia F 2.0, Kuraray Medical Inc., Japan)
ZL	Zirconia (inCoris ZI, Sirona Dental Systems, GmbH, Bensheim, Germany)	Lithium disilicate ceramic (IPS e-max CAD, Ivoclar Vivadent, Schaan, Liechtenstein)		Fusion glass ceramic (IPS e.max CAD Crystall./Connect, Ivoclar Vivadent, Schaan, Liechtenstein)

All finished restorations were luted with zinc phosphate cement (Adhesor; SpofaDental, Czech Republic) under a constant 30 N load. Before mechanical testing, all restorations were incubated in distilled water at 37 °C for 24 h. 

### Failure load testing

To determine the failure load, each specimen was mounted in a universal testing machine (Instron 8801; Instron Ltd, High Wycombe, UK). A compressive force was directed perpendicular to the occlusal surface using a 5.0 mm diameter stainless steel ball at a crosshead speed of 0.5 mm/min. The maximum load at the point of failure was recorded for each sample utilizing the Bluehill software (Instron Ltd).

### Statistical analysis

The power analysis indicated that a sample size of *n* = 12 per group would be sufficient to achieve a 90% power at the 0.05 significance level. This sample size was in accordance with the previous studies in the literature [22, 23].

All statistical evaluations were executed with SPSS version 24.0 (IBM Corp., Armonk, NY, USA). After confirming the homogeneity of variances through Levene test, the data were analyzed with the independent samples t-test. The level of significance was set at *p <* 0.05.

### Fractographic analysis

Following the failure load testing, each specimen was visually examined to determine the failure mode, categorized as chipping, delamination, or bulk fracture. Additionally, the implant-abutment complexes were inspected for secondary complications, such as screw loosening or abutment deformation.

### Finite Element Analysis (FEA)

The 3D geometry of the bone tissue, abutment and implant components was adopted from a previously described and validated model [22]. A four-node tetrahedral meshing approach was applied to the complete system. The crown models compromised 129,559 nodes and 713,234 elements. While loading parameters (a 300 N static vertical load) were consistent with the previous study [22], the mechanical properties of the materials are summarized in Table 3 with their respective references. Von Mises, minimum principal (Pmin) and maximum principal (Pmax) stresses were calculated using Abaqus (version 6.14.1; Dassault Systèmes) to evaluate the biomechanical behavior of the restorations on the implant-abutment-bone complex.

**Table 3:** Mechanical features of the materials used in FEA

Material	Young’s modulus (GPa)	Poisson ratio	References
InCoris ZI	210	0.26	[38]
E.max CAD	95	0.20	[38]
Vita Mark II	45	0.21	[39]
E.max CAD Crystall./Connect	70	0.21	[38]
Panavia F2.0	18.6	0.28	[38]
Implant and abutment	114	0.34	[22]
Cortical bone	13.7	0.3	[22]
Spongious bone	1	0.3	[22]

## Results

The descriptive data, including the mean, standard deviation (SD), and range (minimum to maximum) of the failure loads, are detailed in Table 4. Group ZL exhibited significantly higher failure load values (4649.22 ± 733.42 N) compared to Group ZF (2085.91 ± 555.61 N). One specimen from Group ZL was excluded as abutment deformation prevented further loading and 11 samples were analyzed. According to the independent samples t-test results, a remarkably significant difference existed between the groups (*p* < 0.001).

**Table 4:** Descriptive analysis of the groups

Group	*N*	Mean (*N*)	Standard deviation	Minimum	Maximum
ZF	12	2085,91^a^	555,61	1182,99	3224,60
ZL	11	4649,22^b^	733,42	3351,78	5390,54

Different superscript letters indicate statistically significant differences (*p* < 0.05)

### Fractographic analysis

**Fig. 1:**
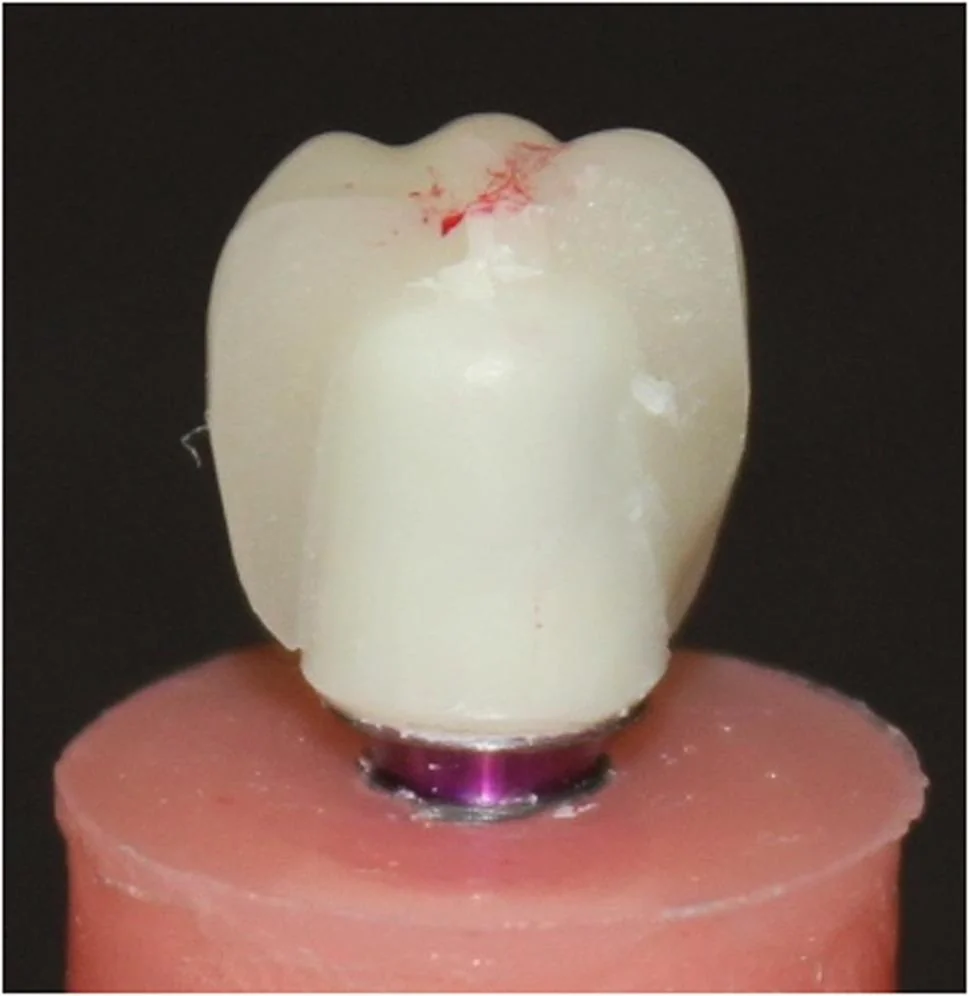
Delamination failure in Group ZF

**Fig. 2:**
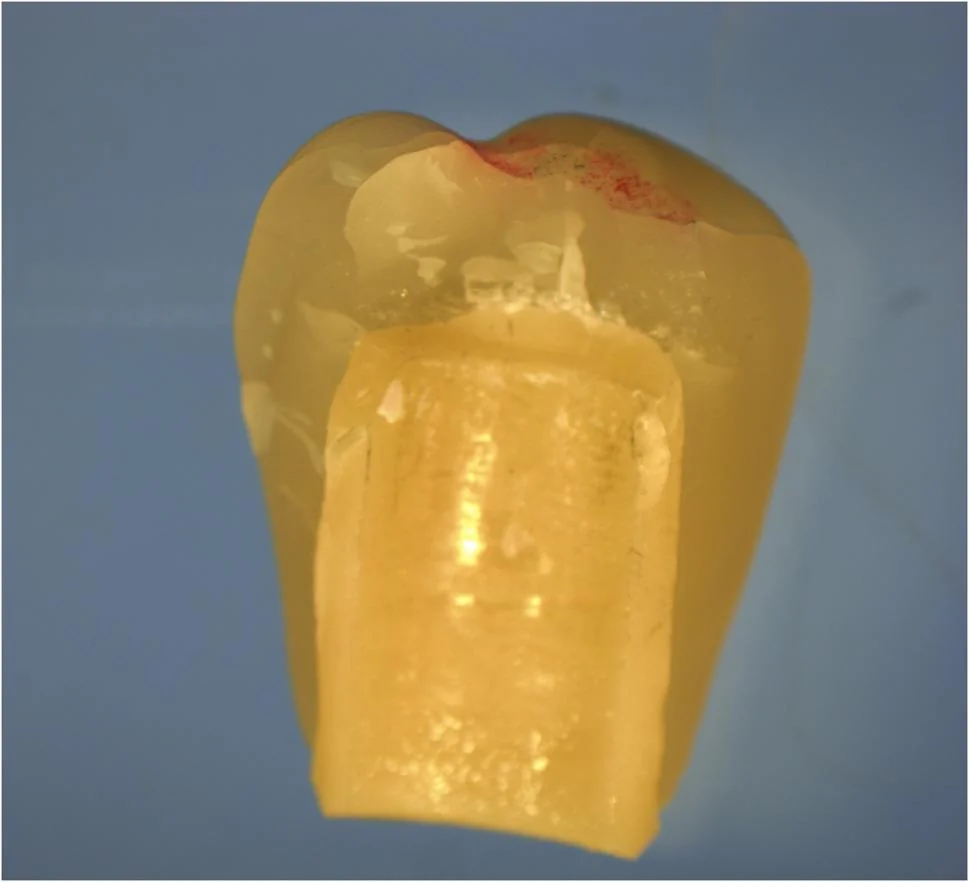
Stereomicroscope image of a specimen of the Group ZL

**Fig. 3:**
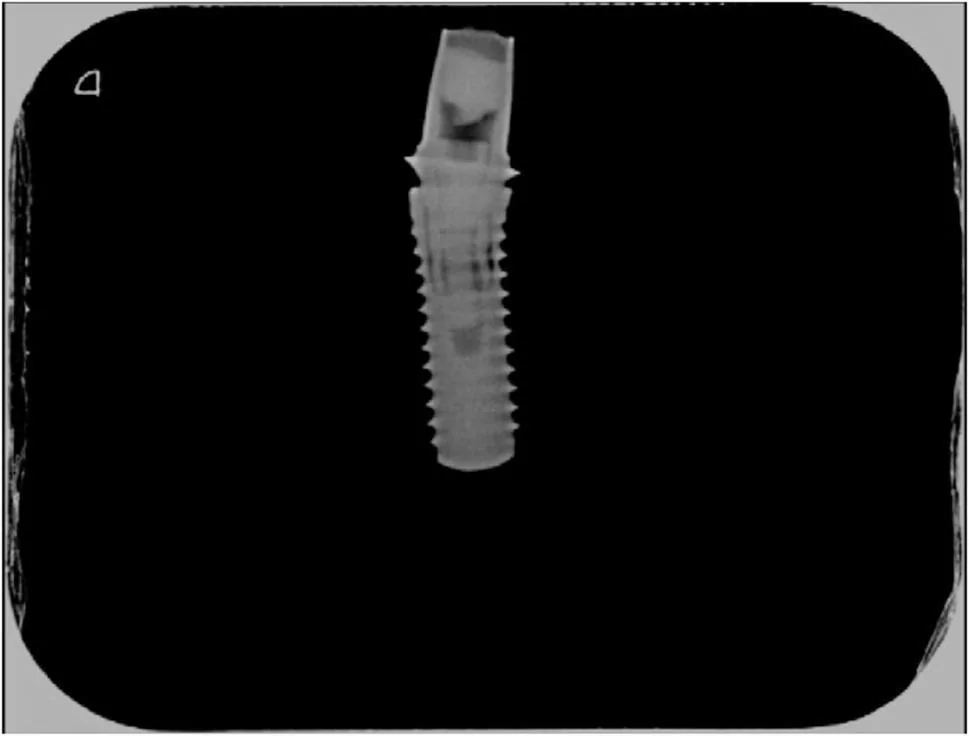
Radiographic view of the abutment deformation in Group ZL

In Group ZF, delamination was observed in all restorations (100%) (Fig. 1). In Group ZL, four restorations exhibited delamination, while the remaining specimens showed bulk fracture (Fig. 2). Regarding the implant-abutment components, 11 abutment in Group ZL was deformed (Fig. 3), and screw loosening was observed in 1 specimen. In contrast, no abutment deformation or screw loosening was detected in Group ZF.

### Finite Element Analysis (FEA)

**Fig. 4:**
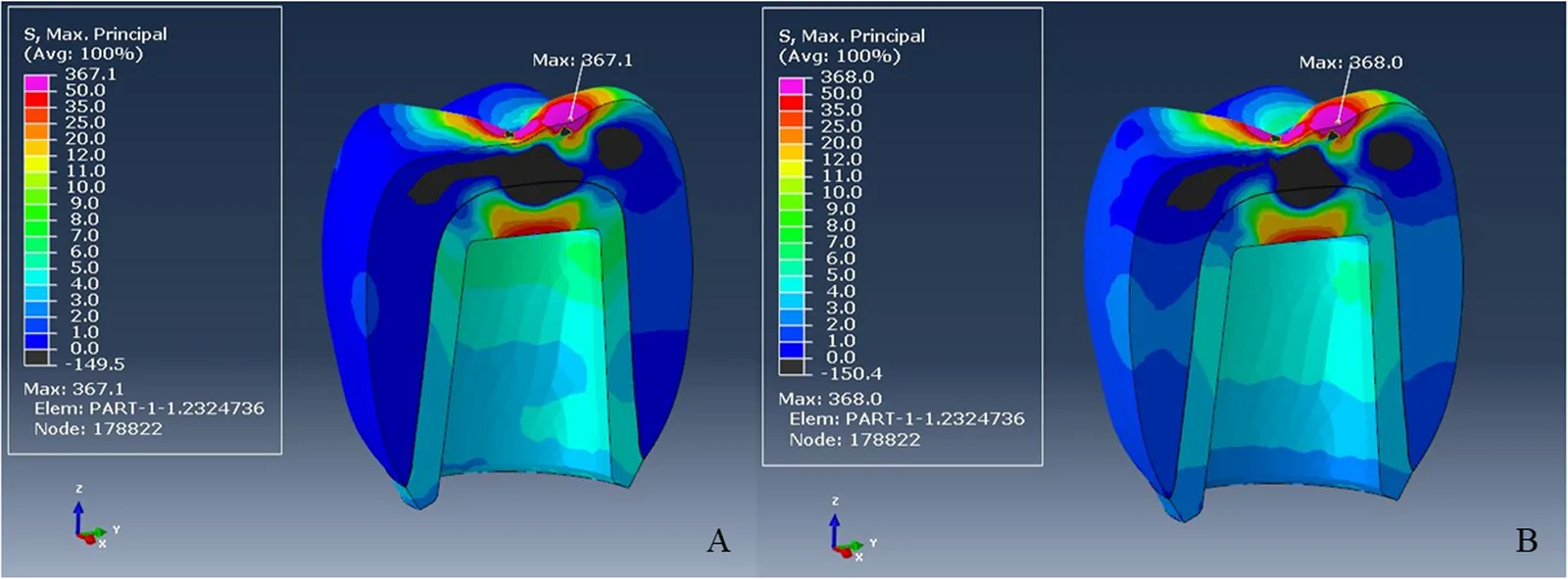
Maximum Principal Stress distributions on crowns: (**A**) Group ZF and (**B**) Group ZL

**Fig. 5:**
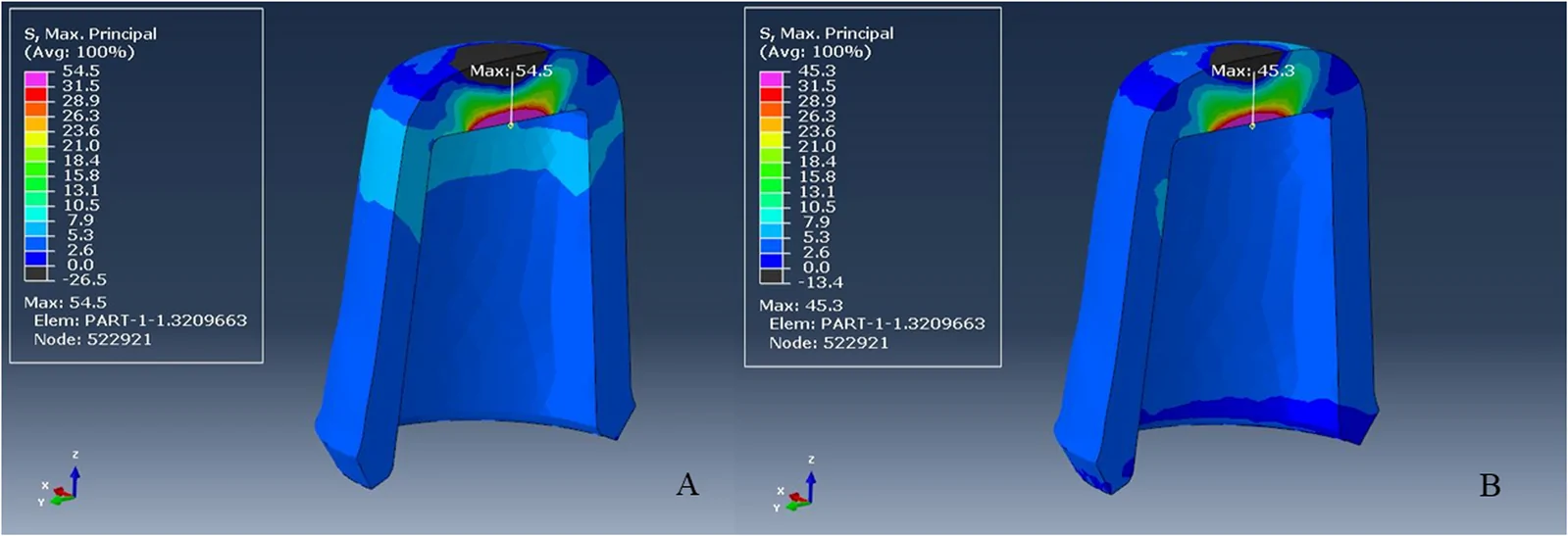
Maximum Principal Stress distributions on zirconia cores: (**A**) Group ZF and (**B**) Group ZL

**Fig. 6:**
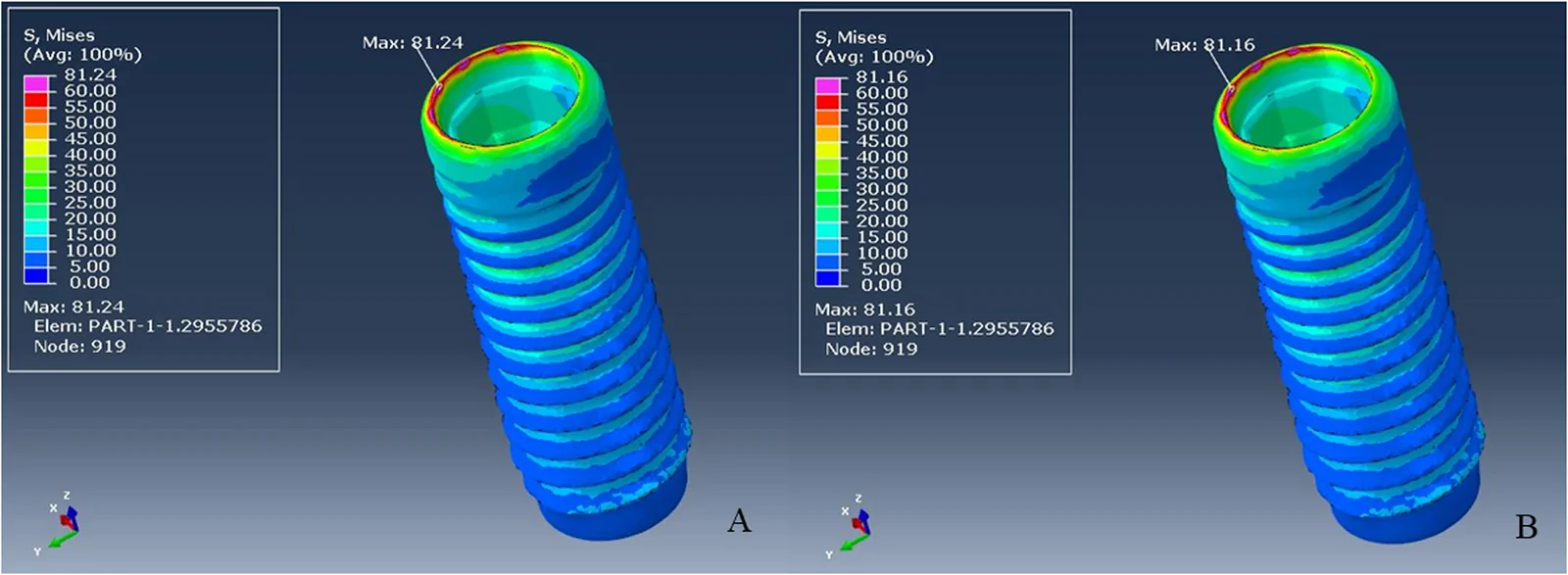
Von Mises Stress distributions on the implant: (**A**) Group ZF and (**B**) Group ZL

**Fig. 7:**
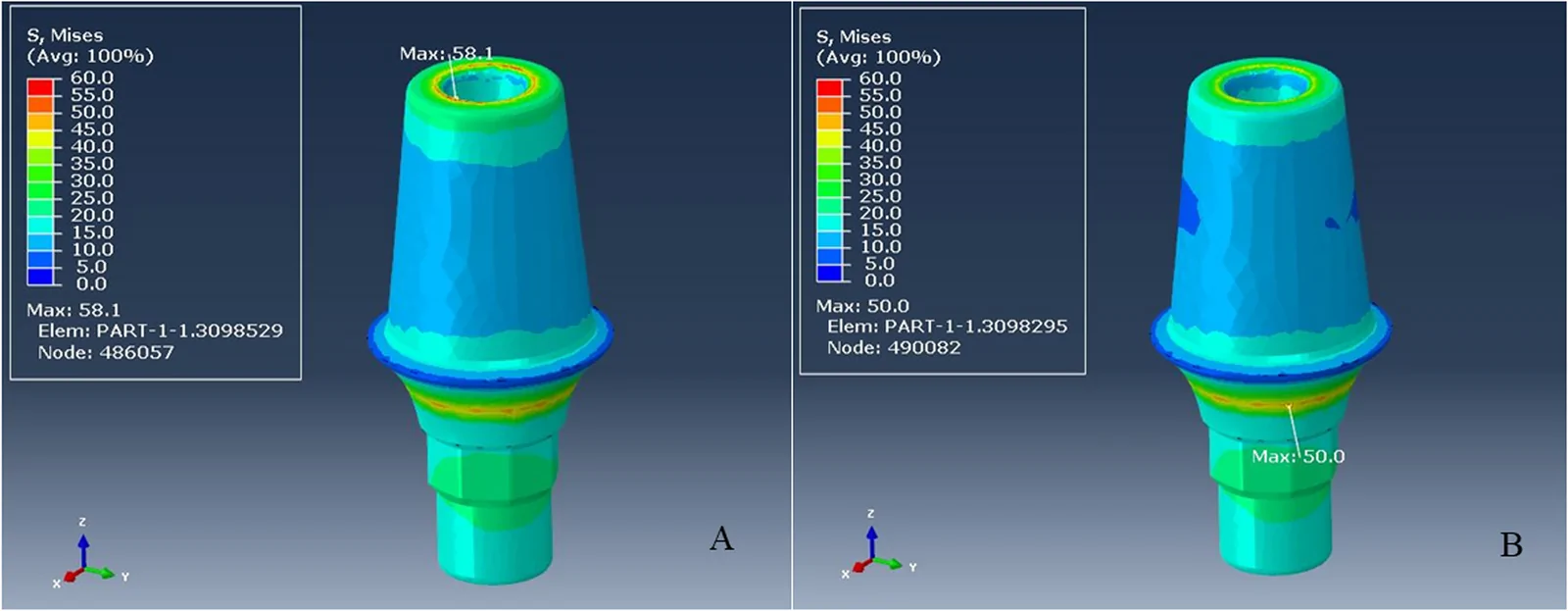
Von Mises Stress distributions on the abutment: (**A**) Group ZF and (**B**) Group ZL

**Fig. 8:**
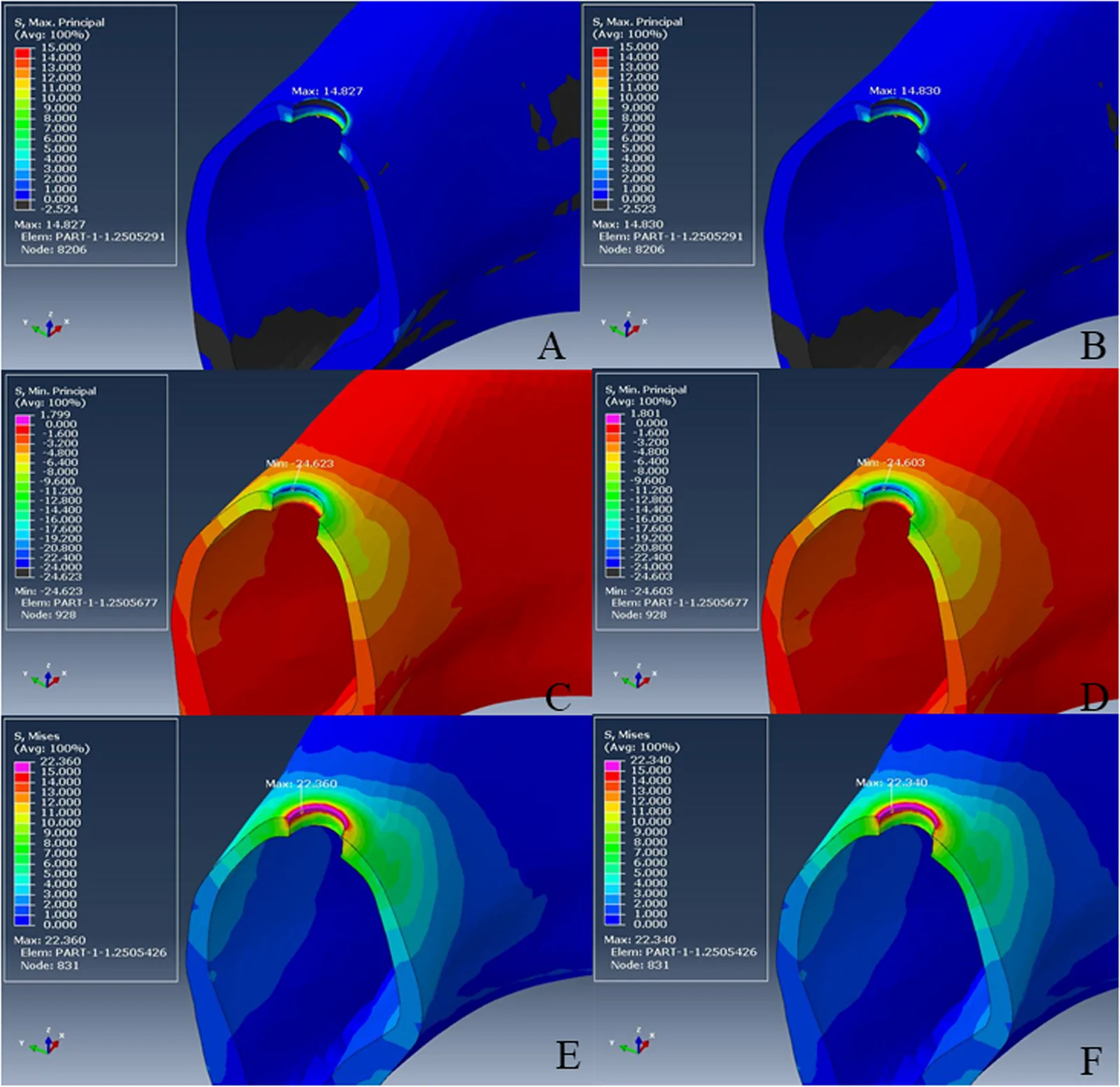
Comparison of stress distributions in the bone for Group ZF (left column) and Group ZL (right column): (**A**, **B**) Maximum Principal Stress; (**C**, **D**) Minimum Principal Stress; (**E**, **F**) Von Mises Stress

The maximum principal stress (Pmax) values were nearly identical for both groups (Group ZF: 367.1 MPa; Group ZL: 368.0 MPa) (Fig. 4a-b). Tensile stresses were primarily concentrated at the occlusal aspect of the core; however, the Pmax value in Group ZF (54.5 MPa) was higher than in Group ZL (45.3 MPa) (Fig. 5a-b). Von Mises stress values were comparatively similar between groups and they were concentrated in the neck of the implants (Fig. 6a-b). In Group ZF, Von Mises stresses on the abutment were concentrated at the most coronal part, whereas in Group ZL, the stresses were located above the platform (Fig. 7a-b). The distribution and values of Von Mises, Pmax and Pmin stresses in the surrounding bone were comparable for both groups (Fig. 8a-f).

## Discussion

The present study demonstrated that CAD-on systems exhibited significantly different effects on failure load, fracture patterns, and the deformation of implant-abutment components. Particularly, the use of LC (Group ZL: 4649.22 N) increased the failure load to more than twice that of the FC (Group ZF: 2085.91 N). Therefore, the null hypothesis was rejected. The higher failure load can be attributed to the superior mechanical properties of LC. The flexural strength values of this material are reported to range from 210 to 360 MPa [24], whereas FC has a lower flexural strength of approximately 136 MPa (Table 1). This difference may also explained by the materials’ microstructure. While FC has a heterogeneous feldspar structure with crystalline phases [25], LC contains a glass matrix reinforced with 70% crystals [26].

The significantly higher failure loads observed in the current study compared to literature, demonstrated the biomechanical advantage of the CAD-on systems. Kok et al. reported a fracture resistance of 2788 N for monolithic LC [1]. In the other studies, failure load value was approximately 3000 N for the same material [22, 27], whereas CAD-on crowns with LC veneer in the present study achieved 4649 N. The same reinforcing effect was also observed for the other group. While previous studies on monolithic FC crowns reported fracture strengths between 851 N and 1267 N [28, 29], in our study this value increased to 2085 N. In another study evaluating monolithic implant-supported crowns, LC (2645 N) exhibited more than twice the fracture strength of FC (1130 N) [23]. This is consistent with our findings, which revealed a similar ratio but nearly twice the absolute failure load values for both materials. The variations in failure load values between these studies may partly originate from differences in the experimental setup, such as the diameter of the loading indenter and aging protocols. While Rosentritt et al. [27] and Doğan et al. [23] employed thermal cycling and mechanical loading to simulate fatigue, the present study utilized a static loading protocol, potentially accounting for the higher failure load values observed. Furthermore, while Kok et al. [1] used a 12-mm loading tip, the current study employed a 5-mm indenter. Despite the smaller contact area of our indenter, which typically concentrates stress, our specimens exhibited significantly higher failure loads. This suggests that the structural reinforcement in our design effectively compensates for the increased local stress. Unlike the aforementioned studies that focused on monolithic crowns where the load is borne by a single material layer, the restorations in the present study utilized a 0.5-mm zirconia core. This framework may act as a high-modulus internal support that redistributes occlusal forces and prevents the veneer from reaching its breaking point prematurely. Such an improvement is critical for long-term clinical success, as chipping has been identified as a primary failure mode in implant-supported crowns with LC cores by Rammelsberg et al. [30]. This reinforced design effectively manages the high stress concentrations, potentially reducing the clinical chipping risks reported in recent systematic review [31].

The variations in failure modes between the two groups can be attributed to the differences in their interfacial bonding mechanisms. In Group ZF, veneer delamination was observed in all specimens (100%), which indicates that the interfacial bond between the FC and the zirconia core is the primary site of failure initiation. The challenge of achieving a stable chemical bond to zirconia, combined with the relatively lower elastic modulus of resin cements, causes stress concentration at the interface and results in the separation of the veneer layer from the core [32]. Consistent with these findings, Contreras et al. reported that resin-cemented group exhibited the lowest performance in their in vitro study on zirconia and FC [33].

In contrast, Group ZL primarily exhibited bulk fractures rather than delamination. This behavior suggests a more homogenous structural integrity, where the restoration functions as a single unit, similar to a monolithic design. This can be attributed to the application of fusion glass ceramic instead of resin cement. The fusion glass ceramic, which has a higher elastic modulus, establishes a more rigid and stable transition between the zirconia core and the LC veneer. By effectively filling structural defects and providing a fused interface [15], it prevents failure from initiating at the bonding site. This mechanical performance aligns with the findings of Nogueira et al., who reported that fusion ceramics ensure superior adhesion to zirconia frameworks for both LC and FC veneers [11]. Similarly, Tomm et al., demonstrated that fused CAD-on systems exhibited fatigue fracture behavior comparable to monolithic systems while significantly decreasing the incidence of delamination [15].

Despite the superior strength of Group ZL, this monolithic-like behavior may have a contradictory impact on the implant-abutment complex. Because Group ZL restorations did not fail at lower loads, the mechanical stress was transferred directly to the underlying components, resulting in screw loosening and abutment deformation. Conversely, the delamination observed in Group ZF may function as a protective mechanism for the implant-abutment connection by absorbing energy through veneer failure; notably, no abutment deformation or screw loosening was detected in this group. These findings suggest that while reinforcing material strength provides durability for the crown, it simultaneously increases the biomechanical risk of irreversible damage to the implant components.

The FEA results provide a biomechanical explanation for these mechanical observations. In Group ZL, Von Mises stresses were transferred further apically and concentrated beneath the abutment platform, which may explain the screw loosening and abutment deformation. Furthermore, tensile stresses were concentrated at the occlusal surface of the core in both groups. The higher Pmax value in Group ZF (54.5 MPa) compared to Group ZL (45.3 MPa) can be explained by increasing differences in the elastic modulus between the veneer and the core (Table 3), which typically triggers higher stress concentrations [34]. This is consistent with previous research [22], which demonstrated that various glass-ceramic cores exhibited lower Pmax values than those reported in the present study due to their lower elastic modulus compared to zirconia. On the other hand, unlike the variations observed in mechanical testing, both groups exhibited similar initial stress distributions within the crown restorations and surrounding bone. In the FEA models, this initial similarity is expected, as both models were evaluated under the same static load to assess initial stress patterns. However, during mechanical test, Group ZL reached significantly higher ultimate failure loads, causing excessive energy to be transferred to the implant components. While, the stress concentration at the implant neck observed in the present study is a common finding [22, 35, 36], the different stress pattern located above the platform in Group ZL highlights how the choice of crown material can fundamentally alter stress transmission and failure modes.

According to the validation process of FEA, models should be based on accurate and realistic geometries and be comparable with mechanical tests [37]. In the present study, all components were modeled using original digital data and CAD/CAM designs. Besides, the high stress areas identified in the FEA were consistent with the locations of cracks and delamination, confirming the reliability of biomechanical analysis. Moreover, the different stress patterns on the abutment across the groups emphasized the different load transfer mechanisms observed during failure testing.

The present study has some limitations that should be considered. First, the simulated conditions cannot completely replicate the complex intraoral environment, which includes long-term thermal cycling, pH variations, and cyclic loading. Second, while the FEA models provide useful data, they assume that all materials are perfectly connected and behave uniformly under load, whereas these conditions can be more complex in real life. Furthermore, the present study used a static force, while in vivo occlusal forces are typically dynamic and multi-directional. Therefore, long-term clinical and laboratory studies are necessary to validate these biomechanical findings.

## Conclusions

Within the limitations of this in vitro study, the following conclusions can be drawn:

1. Group ZL exhibited significantly superior failure loads compared to Group ZF.
2. The increased failure load in Group ZL was associated with higher stress transfer to the implant components, possibly increasing the risk of screw loosening and abutment deformation under extreme mechanical loads.
3. Conversely, the veneer delamination observed in Group ZF could act as a protective failure mode, potentially reducing the risk of irreversible damage to the implant-abutment complex by failing at lower mechanical loads.
4. Although the FEA revealed that different veneer ceramics did not alter the stress distribution across the implant and the bone, the selection of restorative system should ideally balance high fracture resistance with the biomechanical safety of the implant components.

## Data Availability

All data generated or analysed during this study are included in this published article.

## Abbreviations

3D: Three-dimensional
CAD/CAM: Computer aided design/computer aided manufacturing
CAD-on: CAD/CAM-based veneering technique
FEA: Finite element analysis
FC: Feldspathic ceramic
LC: Lithium disilicate ceramic
Pmax: Tensile strength
Pmin: Compressive strength
